# Difference of compliance rates for the recommendations in Japanese Guideline on Febrile Neutropenia according to respondents’ attributes: the second report on a questionnaire survey among hematology-oncology physicians and surgeons

**DOI:** 10.1007/s00520-022-06834-9

**Published:** 2022-01-29

**Authors:** Nobu Akiyama, Takuho Okamura, Minoru Yoshida, Shun-ichi Kimura, Shingo Yano, Isao Yoshida, Hitoshi Kusaba, Kosuke Takahashi, Hiroyuki Fujita, Keitaro Fukushima, Hiromichi Iwasaki, Kazuo Tamura, Toshiaki Saeki, Yasushi Takamatsu, Sadamoto Zenda

**Affiliations:** 1 Department of Internal Medicine, School of Medicine, Teikyo University, Kaga 2-11-1, Itabashi ward, Tokyo, 173-8605 Japan; 2 Department of Breast Surgery, School of Medicine, Tokai University, Isehara, Kanagawa Japan; 3 Fourth Department of Internal Medicine, Teikyo University Hospital Mizonokuchi, Kawasaki, Kanagawa Japan; 4 Division of Hematology, Jichi Medical University Saitama Medical Center, Saitama, Saitama Japan; 5 Division of Clinical Oncology and Hematology, The Jikei University School of Medicine, Minato, Tokyo, Japan; 6 Department of Hematologic Oncology, National Hospital Organization Shikoku Cancer Center, Matsuyama, Ehime, Japan; 7 Department of Comprehensive Clinical Oncology, Faculty of Medical Sciences, Kyushu University, Fukuoka, Fukuoka Japan; 8 Department of Respiratory Medicine, Anjo Kosei Hospital, Anjo, Aichi Japan; 9 Department of Hematology, Saiseikai Yokohama Nanbu Hospital, Yokohama, Kanagawa Japan; 10 Department of Pediatrics, Dokkyo Medical University, Mibu, Tochigi, Japan; 11 Department of Infection Control and Prevention, Faculty of Medical Sciences, University of Fukui, Eiheiji, Fukui Japan; 12 Professor Emeritus, Fukuoka University, Fukuoka, Fukuoka Japan; 13 Department of Breast Oncology, Saitama Medical University International Medical Center, Hidaka, Saitama Japan; 14 Department of Hematology, Oncology, Endocrinology and Infectious Disease, Fukuoka University, Fukuoka, Fukuoka Japan; 15 Department of Radiation Oncology, National Cancer Center Hospital East, Kashiwa, Chiba Japan

**Keywords:** Febrile neutropenia, Female physician, Guidelines, Supportive care, Chemotherapy, Surveillance

## Abstract

**Purpose:**

The Japanese Society of Medical Oncology (JSMO) published a guideline (GL) on febrile neutropenia (FN) in 2017. This study aims to identify promoting factors and disincentives for complying with GL recommendations according to attributes of doctors providing chemotherapy.

**Methods:**

A questionnaire survey was conducted with SurveyMonkey™ for physician members of the Japanese Association of Supportive Care in Cancer and relevant academic organizations. Each question had four options (always do, do in more than half of patients, do in less than half, do not at all) and a free description form. Responses were analyzed according to the respondents’ attributes.

**Result:**

Seven hundred eighty-eight out of retrieved 801 responses were available for analysis. Multivariable analysis demonstrated that the percentage of GL users was higher among women and Japanese Society of Clinical Oncology members. The overall compliance rate was higher among women, JSMO members, and board-certified medical oncologists. Internists emphasized the significance of collecting blood cultures at FN onset, and surgeons stressed the importance of G-CSF prophylaxis. Hematologists were less likely to adhere to recommendations on risk assessment of FN by the Multinational Association of Supportive Care in Cancer score and administration of gammaglobulin products. However, those are acceptable due to the characteristics of their practice. Eight recommendations had no difference in compliance rates between users and non-users, some of whose statements were ambiguous and discretionary.

**Conclusion:**

Women were more likely to use and adhere to GL. The recommendations should be developed considering the characteristics of specialty and subspecialty and avoiding ambiguity and discretionary statements.

**Supplementary Information:**

The online version contains supplementary material available at 10.1007/s00520-022-06834-9.

## Introduction

We previously published the first report of a questionnaire survey on penetration and application of the Japanese Guidelines (GL) on febrile neutropenia (FN), and revised the second edition published by the Japanese Society of Medical Oncology (JSMO) [1]. It demonstrated that 86.7% of respondents knew and used GL; the medians of the complete and complete plus partial compliance rates, which denote the response of “always do” and “always do” plus “do in more than half of patients” in the questionnaire, were 46.4% (range: 7.0–92.8) and 77.8% (range: 35.4–98.7), respectively, in twenty recommendations. The complete compliance rates were less than 30% in seven recommendations. Some of them are feared to deteriorate the quality of FN management, such as the risk assessment of FN with the Multinational Association of Supportive Care in Cancer (MASCC) score at onset (Q2), administration of therapeutic G-CSF (Q11), and primary prophylactic G-CSF (ppG-CSF)(Q16) (Table 1, Supple Table). It is essential to know facilitating factors and disincentives affecting compliance to promote evidence-based supportive care in cancer. Some of them with low compliance rates appeared to relate to some attributes of the respondents. Link H et al. reported that adherence to guidelines was influenced by doctors’ specialty and clinical experience and chemotherapeutic regimens [2]. This study aims to identify attributes and characteristics of the respondents and features of the recommendations that affected compliance and to propose how to enhance compliance with guidelines.

**Table 1:** Questions

Attributes of responders	Gender, a rank of age, year of graduation of medical school, type of institution, subspecialty, board certifications, affiliated academic societies
Questions on GL	
Q1. Do you know the Clinical Guidelines on Febrile Neutropenia revised 2nd version published from the Japanese Society of Medical Oncology in 2017? Q2. Do you assess the risk of FN with the MASCC score? Q3. Do you take two sets of blood cultures from different body sites at the onset of FN in outpatient care? Q4. Do you take two sets of blood cultures from different body sites at the onset of FN in-hospital care? Q5. Do you take one set of blood cultures from each of a peripheral vein and a CVC, if indwelled? Q6. Do you treat a high-risk FN patient with beta-lactam monotherapy as the first-line therapy? Q7. Do you treat a low-risk FN patient with oral antibacterial as the first-line therapy? Q8. Do you provide outpatient treatment for a low-risk FN patient? Q9. When fever resolves with initial treatment despite persisting neutropenia, do you switch the initial therapy to oral antibacterial or discontinue it? Q10. When the patient’s general condition is stable despite persistent FN over 3–4 days after the first-line therapy initiation, do you continue it? Q11*. Do you administer therapeutic G-CSF to a patient with FN? Q12*. Do you administer intravenous gamma-globulin for a high-risk FN patient? Q13. When a patient indwelled with CVC has FN accompanied by either thrombophlebitis, infectious endocarditis, or positive blood cultures of *Staphylococcus aureus*, *Pseudomonas aeruginosa*, *Bacillus* species, and *Candida* species, do you remove the CVC? Q14*. Do you practice antibacterial prophylaxis for a patient expected with low-grade neutropenia? Q15. Do you use G-CSF as primary prophylaxis in the regimens of FN occurrence of more than 20%? Q16. Do you use G-CSF as primary prophylaxis to a patient with a high risk for developing FN in the regimens of FN occurrence between 10 and 20%? Q17*. Do you use G-CSF as primary prophylaxis in the regimens of FN occurrence of less than 10%? Q18. Do you screen HBV infection, including the measurement of HBs antigen, anti-HBs antibody and anti-HBc antibody before the initiation of cancer chemotherapy? Q19. Do you screen tuberculosis, including chest X-ray examination and history taking of the previous infection and recent contact with the patients before starting chemotherapy? Q20. Do you practice vaccination of influenza for patients receiving cancer chemotherapy? Q21. Do you practice vaccination of *Streptococcus pneumoniae* for patients receiving cancer chemotherapy when they are either between two months and six years of age or older than 65 years old?	

^*^The GL does not recommend those practices

## Materials and methods

### Design of the questionnaire

The questionnaire consisting of twenty-one questions on GL and seven on attributes of respondents was surveyed from March to May 2020 through SurveyMonkey™ for the members of the Japanese Association of Supportive Care in Cancer (JSCC), JSMO, the Japanese Society of Hematology (JSH), and the Japanese Breast Association (JBA) (Table 1). The precise methods of surveillance were reported previously [1]. Each question had four options, (1) always do, (2) do in more than half of patients, (3) do in less than half of patients, and (4) do not at all, and a free description form, except for Q1. The options of Q1 were (1) have a printed GL and apply it to clinical practice, (2) have a download format of GL and apply it to clinical practice, (3) know GL but do not use it, (4) do not know GL. Respondents who chose options 1 and 2 are defined as GL users (USR), while those who chose options 3 and 4 as non-user (NUSR). Each question asked whether a respondent does what GL recommends, except for Q11, 12, 14, and 17. The responses to those questions represent the level of compliance with GL. The answers to “always do” and “do in more than half of patients” suggest complete and partial compliance, respectively. In contrast, Q11, 12, 14, and 17 asked whether a respondent does what GL does not recommend, and the same goes for the answers to “do not at all” and “do in less than half of patients.”

### Parameters for analyses

The specialties of respondents were divided into two categories: physician (PHS), meaning internist, and surgeon (SRG), who traditionally provide chemotherapy in their specialties and manage associated adverse effects, including FN. PHS was further divided into hematologist (HEM) and medical oncologist (ONC), including genuine medical oncologists, pulmonologists, gastroenterologists, pediatricians. SRG was also divided into breast surgeons (BRS) and surgeons other than breast surgery (NBS), including gastroenteric surgeons, thoracic surgeons, gynecologists, urologists. The details of respondents’ specialties were mentioned in the previous report [1]. The analyses included two societies and two certifications related to cancer chemotherapy that may influence the use and compliance. These are JSMO, which focuses on internists, and the Japanese Society of Clinical Oncology (JSCO), which focuses on surgeons, as well as the Board-Certified Medical Oncologist (BCMO) accredited by JSMO and the Board-Certified General Clinical Oncologist (BCGCO) accredited by the Japanese Board of Cancer Therapy.

### Statistical analyses

The collected responses were statistically analyzed with Microsoft Excel, R, EZR [3], Python, and Statcel 3. Microsoft Excel was applied to the aggregation of the collected data and the calculation of frequency. R and EZR were used to cross-tabulation table analyses with Fisher’s exact probability test, Mann–Whitney’s *U* test, correlation analysis, estimation of phi coefficient, binominal logistic linear regression analysis, and multivariable linear regression analysis. Statcel 3, an add-in application of Microsoft Excel, was applied to the Kruskal–Wallis test and Chi-square test. Heatmaps were produced by Python with numpy, pandas, and matplotlib.

## Results

Evaluable seven hundred eighty-eight responses were extracted from collected eight hundred and one responses, excluding those of a pharmacist and twelve physicians who did not manage FN patients in their specialty.

### Characteristics of the respondents associated with GL usage

A hundred and five respondents (13.3%) did not know or use GL. The average complete compliance rates (CCR) in USR and NUSR were 44.5% and 37.5% (*P* < 0.001), and complete plus partial compliance rates (C + PCR) were 77.5% and 64.5% (*P* < 0.001) by Mann–Whitney’s *U* test. The demographics of the respondents were analyzed with cross-tabulation table analysis with Fisher’s exact test and binomial logistic linear regression analysis (Table 2). Fisher’s exact test demonstrated that NUSR were significantly more in males, the age group of equal to or older than 50 years old (age≧50), non-members of JSMO or JSCO, and doctors who were not BCMO under 5% significant level. The male gender weakly correlated to age≧50 with a phi-coefficient of 0.252 (*P* < 0.001). JSMO member (including both USR and NUSR) moderately correlated to BCMO (USR + NUSR) with a phi-coefficient of 0.451 (*P* < 0.001).

**Table 2:** Demographics of respondents and the frequencies of response as “do not know or do not use GL”

Attribute	Characteristics	Cross-tabulation table analysis		Binominal logistic regression analysis	
		No. of the respondents do not/do (% of “do not”)	*P*-value	Odds ratio (95%CI)	*P*-value
Total		105/683 (13.3)			
Gender	Male* Female	92/521 (15.0) 13/162 (7.4)	0.007995	0.435 (0.230–0.823)	0.0105
Years of age	< 50 50 ≦*	52/414 (11.2) 53/269 (16.5)	0.03335	0.769 (0.486–1.220)	0.261
Institution	University hospital Cancer center hospital General hospital Others	32/253 (11.9) 36/266 (11.2) 31/145 (17.6) 6/19 (24.0)	0.07294		
Specialty	Physician* Surgeon	48/374 (11.4) 57/309 (15.6)	0.09283	1.230 (0.681–2.210)	0.495
Subspecialty	Hematologist Medical oncologist	11/107 (9.3) 37/267 (12.2)	0.49560		
	Breast surgeon Surgeons other than breast surgery	45/242 (15.7) 12/67 (15.2)	1.00000		
Japanese Society of Medical Oncology	Member* Non-member	62/491 (11.2) 43/192 (18.3)	0.01134	1.390 (0.791–2.430)	0.255
Japanese Society of Clinical Oncology	Member* Non-member	40/347 (10.3) 65/336 (16.2)	0.01601	1.870 (1.180–2.990)	0.00834
Board-certified medical oncologist	Qualified* Non-qualified	26/251 (9.4) 79/432 (15.5)	0.01594	1.270 (0.721–2.220)	0.412
Board-certified general clinical oncologist	Qualified* Non-qualified	38/275 (12.1) 67/408 (14.1)	0.4548	1.040 (0.666–1.610)	0.875

^*^Explanatory variables in the binominal logistic regression analysis with using GL as an objective variable

Binomial logistic regression analysis was applied to examine the relation between the use of GL as an objective variable and the seven explanatory variables as follows: male, age≧50, PHS, JSMO and JSCO member, BCMO, and BCGCO. The result indicated that the usage was significantly lower in males with an odds ratio = 0.435 (95% confidence interval, CI: 0.230–0.823, *P* = 0.0105) and higher in JSCO members with an odds ratio = 1.870 (95%CI: 1.180–2.990, *P* = 0.00834) when removed impacts of potentially confounding roles of the other six variables. There was no correlation between the two parameters, with a phi-coefficient of 0.0546 (*P* = 0.125).

The frequency of USR over 50 years old were 83.2% (242/291) in men and 87.1% (27/31) in women (*P* = 0.799, Fisher’s exact test) and under 50 were 86.6% (279/322) and 93.8% (135/144) (*P* = 0.0256, Fisher’s exact test), respectively. Percentages of USR in academic societies were as follows: JSMO 88.8%, JSCO 89.7%, JSCC 90.5%, JSH 88.3%, JBA 85.0%, and those were not significantly different (*P* = 0.31, Fisher’s exact test).

### Status of compliance with GL’s recommendations

We analyzed compliance status in six hundred eighty-three USR with the Mann–Whitney’s *U* test, Kruskal–Wallis test, and multivariable linear regression analysis. When compared characteristics in an attribute with a significant level of 5%, CCR was significantly higher in age < 50, PHS, ONC, JSMO members, and BCMO. C + PCR was significantly higher in females, PHS, JSMO members, and BCMO (Table 3). Female doctors accounted for 18.2% (77/422) and 26.8% (98/366) in PHS and SRG (*P* = 0.0046, Fisher’s exact test). Gender did not correlate with PHS with correlation coefficients of 0.122 (*P* = 0.0134). Ten pediatricians participated in the survey. Seven of them were USR. Their CCR and C + PCR were markedly low as 21.4% and 47.9%, respectively.

**Table 3:** CCR and C + PCR in GL users

Attribute	Characteristics		CCR		C + PCR	
		No.	No. (%)	*P*-value	No. (%)	*P*-value
Overall		683	8.9 (44.5)		15.5 (77.5)	
Gender	Male Female	521 162	8.8 (44.0) 9.1 (45.5)	0.5188	15.3 (76.5) 16.0 (80.0)	0.0117
Years of age	< 50 50 ≦	414 269	9.1 (45.5) 8.6 (43.0)	0.0486	15.7 (78.5) 15.3 (76.5)	0.0660
Institution	University hospital Cancer center hospital General hospital Others	253 266 145 19	9.1 (45.5) 8.7 (43.5) 8.7 (43.5) 9.4 (47.0)	0.3257*	15.7 (78.5) 15.4 (77.0) 15.3 (76.5) 14.6 (73.0)	0.0649*
Specialty	Physician* Surgeon	374 309	9.1 (45.5) 8.6 (43.0)	0.0308	15.8 (79.0) 15.1 (75.5)	0.0108
Subspecialty	Hematologist Medical oncologist	107 267	8.3 (41.5) 9.4 (47.0)	0.0021	15.6 (78.0) 15.8 (79.0)	0.2735
	Breast surgeon Surgeons other than breast surgery	242 67	8.7 (43.5) 8.5 (42.5)	0.7257	15.2 (76.0) 15.1 (75.5)	0.8217
Japanese Society of Medical Oncology	Member Non-member	491 192	9.1 (45.5) 8.4 (42.0)	0.0066	15.8 (79.0) 14.8 (74.0)	< 0.0001
Japanese Society of Clinical Oncology	Member Non-member	347 336	8.9 (44.5) 8.9 (44.5)	0.9967	15.3 (76.5) 15.7 (78.5)	0.0789
Board-certified medical oncologist	Qualified Non-qualified	251 432	9.4 (47.0) 8.6 (43.0)	0.0016	16.1 (80.0) 15.1 (75.5)	< 0.0001
Board-certified general clinical oncologist	Qualified Non-qualified	275 408	8.9 (44.5) 8.9 (44.5)	0.9905	15.6 (78.0) 15.5 (77.5)	0.4443

Mann–Whitney’s *U* test, significance level both sides: *P* < 0.05

^*^Kruskal–Wallis test, significance level upper side: *P* < 0.05

The multivariable analysis was applied to examine the association between CCR and C + PCR as objective variables and the seven explanatory variables of male, age≧50, PHS, members of JSMO and JSCO, BCMO, and BCGCO. CCR was significantly higher in BCMO when removed impacts of potentially confounding roles of the other six variables. And C + PCR was higher in JSMO members and BCMO and lower in males, significantly (Table 4).

**Table 4:** Multivariable linear regression analyses on CCR and C + PCR

	CCR				C + PCR			
Explanatory variables	Estimated regression coefficient	Standard error	*t*-value	*P*-value	Estimated regression coefficient	Standard error	*t*-value	*P*-value
Intercept	43.4687	1.7196	25.2780	< 0.0001	76.1407	1.3979	54.4682	< 0.0001
Gender (male, 1; female, 0)	− 1.3693	1.5389	− 0.8898	0.374	− 4.0355	1.2510	− 3.2259	0.00132
Years of age (≧50, 1; < 50, 0)	− 1.0499	1.3858	− 0.7576	0.449	− 0.1955	1.1265	− 0.1735	0.862
Specialty (physician, 1; surgeon, 0)	− 0.7597	1.7435	− 0.4357	0.663	0.0759	1.4173	0.0536	0.957
Japanese Society of Medical Oncology (member, 1; non-member, 0)	2.4913	1.7684	1.4088	0.159	3.3286	1.4375	2.3155	0.0209
Japanese Society of Clinical Oncology (member, 1; non-member, 0)	− 0.1695	1.3436	− 0.1261	0.900	1.5020	1.0922	1.3752	0.170
Board-certified medical oncologist (qualified, 1; non-qualified, 0)	3.3497	1.5637	2.1422	0.0325	3.5151	1.2711	2.7653	0.00584
Board-certified general clinical oncologist (qualified, 1; non-qualified, 0)	− 0.1976	1.2821	− 0.1542	0.878	0.0718	1.0423	0.0689	0.945

JSMO member (including USR only) was moderately correlated with BCMO (USR only) with a correlation coefficient of 0.456 (*P* < 0.0001) and PHS with a correlation coefficient of 0.583 (*P* < 0.0001). Male did not correlate with JSMO member and BCMO with correlation coefficients of 0.0954 (*P* = 0.0126) and 0.0395 (*P* = 0.302).

### Heatmaps of the CCR and C + PCR

CCR and C + PCR of each characteristic are demonstrated in Figure as heatmaps processed from the data of the supplemental table. Differences of CCR and C + PCR between two characteristics in an attribute were analyzed with cross-tabulation table analyses with Fisher’s exact test. The results are indicated in the supplemental table. Except for the NUSR line, all the data are derived from those of USR.

## Discussion

In the previous report, we demonstrated and discussed the results of question-based analysis [1]. This report discusses the results based on the respondent’s attribute and explores clues to interventions corresponding to the characteristics of each attribute. Five discussion points were extracted from the findings as follows.

### Higher rates of USR and compliance in female doctors

Gender was one of the two significant influencing factors on GL usage revealed by the binomial logistic regression analysis. And it was one of the three explanatory variables extracted in the multivariable regression analysis on C + PCR. The percentage of USR and C + PCR in females was significantly higher than in males (Tables 2 and 3). The respondent consisted of 613 (77.8%) men and 175 (22.2%) women (Table 2). According to the Ministry of Health, Labor, and Welfare’s 2018 data, female doctors comprise 21.9% of the total doctors in Japan, and the ratio decreases to 13.2% only for those over 50 years old. The percentage of women among respondents and the correlation between male and age≧50 are considered reflections of the current demographic of doctors in Japan (https://www.mhlw.go.jp/toukei/saikin/hw/ishi/18/dl/kekka-1.pdf). Therefore, the results of the survey are regarded as free of methodological gender bias. It is reasonable to conclude that female doctors use and comply with GL more than male doctors.

The higher compliance rate among female doctors appears to be a common feature worldwide and across specialties compared to male doctors [4, 5]. Tsugawa Y et al. described in their report that female physicians might be more likely to adhere to guidelines. And they also referred to the better practice patterns of female physicians, including providing preventive care, using patient-centered communication, performing as well or better on standardized examinations, and providing psychosocial counseling to their patients. Besides, they reported that mortality and readmission rates for elderly hospitalized patients treated by female internists were lower than those by male internists as the results of their investigation. We could not find any description of why female doctors are more compliant with guidelines in previous investigations. It is out of reach to speculate the difference between both genders. Although it is difficult to intervene in gender-specific characteristics, the publication of these results may help change male doctors’ attitudes about GL use and compliance.

### The influence of academic societies and board-certification

JSCO members were extracted as one of the two significant influencing factors on GL usage by the binomial logistic regression analysis (Table 2). JSCO members were not invited to the study directly. However, almost all of them (99.3%) belonged to at least one of the four aforementioned societies. We cannot clearly explain the association between JSCO membership and GL use, but this is probably due to the particular relation between JSCO and the other societies in this survey rather than the membership itself. JSMO members and BCMO were each identified as one of the three significant explanatory variables on C + PCR by the multivariable regression analysis (Table 4). The impact of these characteristics on C + PCR is considered smaller than gender according to the estimated regression coefficients as around 3.

The pattern of difference in compliance with each recommendation between JSMO members and non-members is almost identical to that between PHS and SRG, as discussed below (Figure and Supple Table). This is presumably due to a moderate correlation between JSMO members and PHS. The CCR for all questions except Q16 in BCMO and C + PCR were significantly higher than or equal to those in non-BCMO. In particular, C + PCR of Q2, the risk assessment for FN at the onset with MASCC score, was 64.5%, the highest among other attributes. These findings mean that BCMO is considered a reliable indicator of high GL compliance. Alternatively, BCGCO seems to have little effect on compliance.

These results may be explained by the fact that JSMO issued GL and accredits BCMO. JSMO’s educational role may function to promote evidence-based clinical practice in compliance with GL. It is necessary to send a strong message to doctors providing chemotherapy about GL compliance through not only JSMO but also other relevant academic societies. Besides, patient education on FN and infections associated with chemotherapy is an effective promotion measure, and academic societies should take the lead in this effort.

### Differences in compliance rate according to specialty and subspecialty

A survey of G-CSF adherence in Germany revealed that specialty influenced doctors’ adherence to guidelines. Pulmonologists were less adherent to GL than other specialists, including hematologists-oncologists and gynecologists, though the cause was uncovered [2]. In such a case, it is assumed that there is some attribute-related barrier to compliance.

**Fig. 1:**
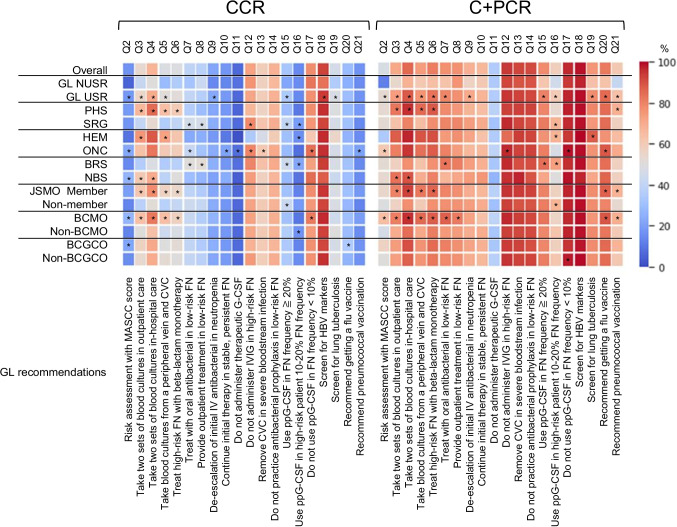
Heatmaps of the CCR and C + PCR for each recommendation according to the attributes and characteristics of responders. *, significantly higher than the counterpart. NUSR, non-user; USR, user; PHS, physician; SRG, surgeon; HEM, hematologist; ONC, medical oncologist; BRS, breast surgeon; NBS, surgeons other than breast surgery; JSMO, Japanese Society of Medical Oncology; BCMO, board-certified medical oncologist; BCGCO, board-certified general clinical oncologist

Between PHS and SRG, the overall CCR and C + PCR of PHS were higher as 45.5% vs. 43.0% (*P* = 0.0308) and 79.0% vs. 75.5% (*P* = 0.0108) (Table 3). The CCR and C + PCR of Q3-6 were significantly higher in PHS, while so were those of Q16 in SRG (Fig. 1 and Supple Table). The CCR of Q3 and 4 (strong recommendations) in PHS vs. SRG were 66.8% vs. 41.4% (*P* < 0.001) and 84.8% vs. 53.4% (*P* < 0.001). The C + PCR of Q16 (weak recommendation) in PHS vs. SRG were 54.0% and 68.3% (*P* < 0.001). The findings indicate that PHS places more importance on blood culture collection than SRG. SRG is more likely to emphasize the prevention of FN development than PHS. Since 78.3% of SRG were BRS, the pattern of compliance in SRG reflected BRS as mentioned below.

Between HEM and ONC, the overall CCR of HEM was lower as 41.5% vs. 47.0% (*P* = 0.0021) and the C + PCR was comparable as 78.0% vs. 79.0% (*P* = 0.2735) (Table 3). The C + PCR for Q2 and 12 (weak recommendations) were significantly lower in HEM (34.5% and 85.0% vs. 61.0% and 96.6%, both *P* < 0.001) (Fig. 1 and Supple Table). In hematologic malignancies, chemotherapy frequently causes severe neutropenia and immunocompromised condition with hypogammaglobulinemia. Therefore, the patients are generally at high risk when FN develops and more require intravenous gammaglobulin than in the other specialties. These are probable reasons for low compliance with Q2 and 12 in HEM, which are features of their practice and should not be seen as problems. Other HEM’s characteristics were more screening for lung tuberculosis and less vaccination for influenza. These may be related to a high prevalence of immunocompromised patients in hematologic diseases. The C + PCR of Q16 in HEM vs. ONC was 68.2% vs. 48.3% (*P* < 0.001). The disincentive for Q16 in ONC is considered to reflect the economic issues indicated in the free comments reported previously [1].

The overall CCR and C + PCR were markedly low in pediatricians. It was probably due to GL recommendations mainly based on the evidence of studies in adult patients.

Between BRS and NBS, the overall CCR and C + PCR were not different (Table 3). However, the patterns of compliance with each recommendation were slightly different. BRS took two sets of blood culture samples considerably less than NBS (CCR: Q3: 36.0% vs. 61.2%, *P* < 0.001; Q4: 48.8% vs. 70.1%, *P* = 0.002) (Fig. 1 and Supple Table). The CCR and C + PCR of Q15 (strong recommendation) were higher in BRS as 43.8% vs. 28.4% (*P* = 0.025) and 84.7% vs. 71.6% (*P* = 0.014). Those of Q16 were 22.3% vs. 9.0% (*P* = 0.014) and 73.1% vs. 50.7% (*P* < 0.001). In general, FN in breast cancer is as frequent and severe as other solid tumors such as lung cancer and gynecological malignancies [6, 7]. Some of the chemotherapy regimens in breast cancer are moderate to high risk for developing FN, and BRS emphasizes ppG-CSF since they treat patients’ ambulatory. Consequently, they have few opportunities to experience bloodstream infection in FN, which may have contributed to the low compliance rate with the recommendations on blood cultures.

There are reportedly seven barriers against physicians’ adherence to practice guidelines: lack of awareness, lack of familiarity, lack of agreement, lack of outcome expectancy, lack of self-efficacy, lack of motivation, and external barriers, including patient factors, guideline factors, and environmental factors [8]. These findings suggest that the specificity of specialty and subspecialty may be another barrier that should be considered when developing multidisciplinary guidelines.

### Low compliance rates about collecting blood culture

The CCR and C + PCR of Q3 and 4 were significantly lower in SRG than PHS, further in BRS than NBS, as mentioned above (Fig. 1 and Suppl Table). HEM marked the highest CCR and C + PCR, which are comparable to the previous report of Kimura S et al. indicating that 92% of HEM collected two sets of blood culture at FN onset in acute myeloid leukemia [9]. The incidence of bloodstream infection in FN was 8.3–11.8% in acute myeloid leukemia and 3% in non-Hodgkin lymphoma reportedly [10–12]. This is the reason why HEM almost routinely takes blood cultures in FN. That in FN of solid tumors has been unknown because of very low incidence. Blood cultures are essential for diagnosing bloodstream infections, but false positives can harm patients through unnecessary antibiotics and hospitalization and cost medical resources humanly and financially. Considering those matters, BRS may dare not to take blood cultures in low-risk FN patients. Previous studies have demonstrated that an algorithm to reduce the overuse of blood cultures effectively prevents unnecessary blood cultures [13, 14]. In the algorithm, immunocompromised patients were excluded. However, if the pretest probability is sufficiently low, it may be safely omitted even in patients receiving chemotherapy. Whether it is possible to skip the collection of blood cultures from low-risk FN patients is an emerging issue that should be investigated.

### Recommendations with no different compliance between USR and NUSR

We noted the difference in compliance between USR and NUSR. C + PCR was significantly higher in USR in twelve recommendations (Fig. 1 and Supple Table). It is considered fairly reasonable. However, the remaining eight recommendations, Q8, 10–14, 17, and 18, had no difference in compliance between them. The reasons are probably at least one of the following: (1) a recommendation is challenging or controversial to implement in clinical practice or requires a change in the inertia of conventional practice, (2) a recommendation should or should not be implemented as a matter of course regardless of GL, or (3) a recommendation is highly discretionary to doctors due to ambiguous and non-specific expression. Q11 falls into the first category. GL weakly recommends not to use therapeutic G-CSF but suggests using it in case of expecting deterioration. A meta-analysis has demonstrated that therapeutic G-CSF does not improve survival but shortens the duration of neutropenia and makes faster recovery from fever [15]. Therefore, it is acceptable for doctors to use therapeutic G-CSF in FN patients as long as it does not harm them. The barriers to compliance are probably due to the conflicting content and the ambiguous standard of decision-making [8]. Q18 falls into the second category. The screening of hepatitis B virus (HBV) makers is an essential examination to prevent de novo hepatitis B that potentially progresses to fatal fulminant hepatitis [16]. Overlooking it may cause the doctor to be sued. That is the driving force to adhere to the recommendation at the C + PCR of 99.0% and 97.1% for USR and NUSR. Q12–14 and 17 are also allocated to the second category. Except for Q13, they commonly recommend not doing something that is considered of little benefit. Q8 and 10 fall into the third category. Considering the low compliance rate of risk assessment of FN by the MASCC score (Q2), the ambulatory treatment of FN patients is considered at doctors’ discretion rather than based on the GL. About Q10, although GL weakly recommends continuing the initial therapy in stable and persistent FN, the definition of “stable” is unclear. And we sometimes experience a rapid aggravation of a stable FN patient to fatal illness. Whether implementing or not those recommendations is likely to be left to the doctor’s discretion. The influence of those recommendations on doctors’ clinical practice may have been limited.

Grol R et al. reported lower compliance rates in the recommendations that were vague and non-specific or that demanded a change in the existing practice routine [17, 18]. Recommendations need to be as free as possible from ambiguous and non-specific expressions or expressions that allow for great discretion by the attending physician.

This study clarified the difference in compliance among attributes and the characteristics of recommendations that affected compliance. These results may provide useful suggestions for revising GL. This study is expected to help other researchers develop and evaluate guidelines. Meanwhile, questionnaires are quick, labor-saving, low-cost, and can be applied for many recommendations at once, though their accuracy varies. Therefore, these results could deviate from the actual practices.

## Supplementary Information

Below is the link to the electronic supplementary material.

Supplementary file1 (PDF 155 KB)

## Data Availability

Not applicable.

## Code availability

The R and Python codes used in this study were not created specifically for this study but were those commonly used for statistical analysis. Therefore, there is nothing, in particular, that should be deposited as code.
