# Supplementary material for: Difference of compliance rates for the recommendations in Japanese Guideline on Febrile Neutropenia according to respondents’ attributes: the second report on a questionnaire survey among hematology-oncology physicians and surgeons
Source: Support Care Cancer. 2022 Jan 29;30(5):4327–36. doi: 10.1007/s00520-022-06834-9 (PMC8942955; doi:10.1007/s00520-022-06834-9)
Supplement: Supplementary file 1 — Supplementary file1 (PDF 155 KB) [file 520_2022_6834_MOESM1_ESM.pdf]

Supplemental table. CCR and C+PCR of each characteristic and the difference between characteristics, Fisher's exact test.

CCR(%)		Q2	Q3	Q4	Q5	Q6	Q7	Q8	Q9	Q10	Q11	Q12	Q13	Q14	Q15	Q16	Q17	Q18	Q19	Q20	Q21
Overall		16.5	53.6	67.9	48.5	49.2	39.7	37.8	22.1	17.9	7.0	72.7	59.4	68.4	32.7	14.6	77.8	92.8	46.4	29.2	16.8
GL use	NUSR	5.7	41.9	50.5	28.6	35.2	32.4	42.9	14.3	13.3	8.6	76.2	58.1	66.7	21.9	15.2	82.9	83.8	35.2	24.8	15.2
	USR	18.2	55.3	70.6	51.5	51.4	40.8	37.0	23.3	18.6	6.7	72.2	59.6	68.7	34.4	14.5	77.0	94.1	48.2	29.9	17.0
	P-value	0.001	0.010	<0.001	<0.001	0.002	0.099	0.253	0.043	0.190	0.492	0.391	0.772	0.681	0.011	0.841	0.180	<0.001	0.013	0.284	0.656
Specialty	PHS	19.0	66.8	84.8	64.2	60.4	36.6	29.4	22.5	18.2	6.1	66.8	57.0	67.6	29.4	10.4	78.1	95.7	49.5	28.9	19.0
	SRG	17.2	41.4	53.4	36.2	40.5	46.0	46.3	24.3	19.1	7.4	78.6	62.8	69.9	40.5	19.4	75.7	92.2	46.6	31.1	14.6
	P-value	0.536	<0.001	<0.001	<0.001	<0.001	0.014	<0.001	0.577	0.760	0.502	0.001	0.122	0.527	0.003	0.001	0.468	0.071	0.456	0.533	0.126
Subspecialty	HEM	9.3	75.7	88.8	72.9	62.6	23.4	24.3	18.7	10.3	1.9	34.6	46.7	59.8	30.8	15.9	61.7	96.3	55.1	26.2	15.0
	ONC	22.8	63.3	83.1	60.7	59.6	41.9	31.5	24.0	21.3	7.9	79.8	61.0	70.8	28.8	8.2	84.6	95.5	47.2	30.0	20.6
	P-value	0.003	0.022	0.170	0.032	0.584	0.001	0.170	0.269	0.012	0.031	<0.001	0.011	0.050	0.701	0.039	<0.001	0.744	0.165	0.464	0.003
	BRS	14.5	36.0	48.8	35.5	37.6	50.4	51.7	25.2	17.8	8.3	79.8	64.0	70.7	43.8	22.3	73.1	93.4	47.5	31.4	14.5
	NBS	26.9	61.2	70.1	38.8	50.7	29.9	26.9	20.9	23.9	4.5	74.6	58.2	67.2	28.4	9.0	85.1	88.1	43.3	29.9	14.9
	P-value	0.017	<0.001	0.002	0.622	0.067	0.003	<0.001	0.466	0.260	0.296	0.365	0.381	0.581	0.025	0.014	0.053	0.149	0.538	0.808	0.924
JSMO	Member	20.0	62.1	78.0	57.0	55.2	39.7	35.2	23.0	18.9	6.7	72.1	59.1	68.8	31.2	12.8	77.8	94.9	48.1	30.5	18.5
	Non-member	13.5	38.0	51.6	37.5	41.7	43.8	41.7	24.0	17.7	6.8	72.4	60.9	68.2	42.7	18.8	75.0	92.2	48.4	28.1	13.0
	P-value	0.060	<0.001	<0.001	<0.001	0.001	0.335	0.118	0.793	0.710	0.981	0.938	0.654	0.877	0.004	0.053	0.434	0.173	0.930	0.534	0.090
BCMO	Qualified	27.1	63.7	82.5	64.1	57.8	42.2	36.3	26.3	19.5	6.8	72.9	57.8	69.3	31.1	10.0	81.7	96.4	45.0	30.7	19.9
	Non-qualified	13.0	50.5	63.7	44.2	47.7	40.0	37.5	21.5	18.1	6.7	71.8	60.6	68.3	36.3	17.1	74.3	92.8	50.0	29.4	15.3
	P-value	<0.001	0.001	<0.001	<0.001	0.011	0.575	0.745	0.155	0.635	0.976	0.747	0.460	0.778	0.162	0.010	0.030	0.063	0.209	0.725	0.119
BCGCO	Qualified	22.2	57.5	70.9	49.8	52.4	40.4	35.3	24.7	18.2	7.3	68.4	60.4	67.3	34.2	13.1	77.1	92.0	46.9	34.9	17.1
	Non-qualified	15.4	53.9	70.3	52.7	50.7	41.2	38.2	22.3	18.9	6.4	74.8	59.1	69.6	34.6	15.4	77.0	95.6	49.0	26.5	16.9
	P-value	0.026	0.362	0.874	0.460	0.676	0.832	0.432	0.462	0.820	0.645	0.068	0.735	0.519	0.919	0.392	0.968	0.050	0.588	0.021	0.951

C+PCR(%)		Q2	Q3	Q4	Q5	Q6	Q7	Q8	Q9	Q10	Q11	Q12	Q13	Q14	Q15	Q16	Q17	Q18	Q19	Q20	Q21
Overall		47.7	71.4	85.9	74.4	81.3	77.2	77.0	69.0	66.9	35.4	94.3	92.3	91.5	78.2	58.5	96.6	98.7	76.0	82.2	61.0
GL use	NUSR	12.4	54.3	68.6	61.0	64.8	62.9	76.2	57.1	61.0	34.3	92.4	89.5	89.5	61.9	45.7	94.3	97.1	63.8	62.9	41.9
	USR	53.1	74.1	88.6	76.4	83.9	79.4	77.2	70.9	67.8	35.6	94.6	92.7	91.8	80.7	60.5	96.9	99.0	77.9	85.2	64.0
	P-value	<0.001	<0.001	<0.001	0.001	<0.001	<0.001	0.826	0.005	0.166	0.797	0.365	0.260	0.436	<0.001	0.004	0.166	0.118	0.002	<0.001	<0.001
Specialty	PHS	53.5	82.9	95.5	85.8	88.8	78.9	75.4	68.2	65.5	38.5	93.3	93.0	92.8	79.7	54.0	97.3	99.2	79.9	85.3	69.0
	SRG	52.8	63.4	80.3	65.0	78.0	79.9	79.3	74.1	70.6	32.0	96.1	92.2	90.6	81.9	68.3	96.4	98.7	75.4	85.1	57.9
	P-value	0.850	<0.001	<0.001	<0.001	<0.001	0.734	0.228	0.090	0.160	0.079	0.107	0.684	0.304	0.469	<0.001	0.504	0.525	0.155	0.947	0.003
Subspecialty	HEM	34.6	87.9	97.2	89.7	90.7	74.8	77.6	72.0	63.6	35.5	85.0	93.5	92.5	76.6	68.2	94.4	100.0	87.9	76.6	65.4
	ONC	61.0	80.9	94.8	84.3	88.0	80.5	74.5	66.7	66.3	39.7	96.6	92.9	92.9	80.9	48.3	98.5	98.9	76.8	88.8	70.4
	P-value	<0.001	0.107	0.306	0.172	0.465	0.218	0.538	0.320	0.614	0.452	<0.001	0.844	0.903	0.354	<0.001	0.036	0.271	0.015	0.003	0.346
	BRS	52.5	57.9	77.7	63.6	77.3	83.5	81.4	74.0	69.0	33.5	96.3	90.9	90.9	84.7	73.1	95.9	99.2	76.4	84.7	57.9
	NBS	53.7	83.6	89.6	70.1	80.6	67.2	71.6	74.6	76.1	26.9	95.5	97.0	89.6	71.6	50.7	98.5	97.0	71.6	86.6	58.2
	P-value	0.856	<0.001	0.037	0.322	0.561	0.003	0.081	0.913	0.258	0.305	0.776	0.098	0.736	0.014	<0.001	0.302	0.167	0.419	0.706	0.958
JSMO	Member	54.6	80.7	92.9	82.5	86.6	80.0	77.4	69.7	66.8	37.5	95.1	93.9	92.7	80.4	57.6	96.9	99.0	77.8	87.4	68.0
	Non-member	49.5	57.3	77.6	60.9	77.1	77.6	76.6	74.0	70.3	30.7	93.2	89.6	89.6	81.3	67.7	96.9	99.0	78.1	79.7	53.6
	P-value	0.230	<0.001	<0.001	<0.001	0.002	0.479	0.816	0.266	0.378	0.098	0.328	0.071	0.187	0.811	0.016	0.962	0.978	0.927	0.011	<0.001
BCMO	Qualified	64.5	80.9	95.6	85.7	88.0	87.3	81.7	72.9	63.7	39.8	96.8	93.6	92.0	83.3	56.6	96.8	99.2	76.1	89.2	69.3
	Non-qualified	46.5	70.1	84.5	71.1	81.5	74.8	74.5	69.7	70.1	33.1	93.3	92.1	91.7	79.2	62.7	97.0	98.8	78.9	82.9	60.9
	P-value	<0.001	0.002	<0.001	<0.001	0.031	<0.001	0.037	0.370	0.085	0.076	0.054	0.469	0.867	0.191	0.113	0.897	0.652	0.389	0.025	0.032
BCGCO	Qualified	57.1	74.5	90.5	76.7	85.5	81.1	77.5	72.4	69.1	33.1	93.8	92.7	90.9	79.3	58.5	95.3	98.2	78.2	85.8	67.6
	Non-qualified	50.5	73.8	87.3	76.2	82.8	78.2	77.0	69.9	66.9	37.3	95.1	92.6	92.4	81.6	61.8	98.0	99.5	77.7	84.8	61.5
	P-value	0.090	0.822	0.185	0.880	0.363	0.358	0.880	0.479	0.550	0.265	0.469	0.969	0.486	0.447	0.399	0.045	0.091	0.881	0.714	0.102

Gray box: *P*-value less than 0.05

NUSR: non-user, USR: user, PHS: physician, SRG: surgeon, HEM: hematologist, ONC: medical oncologist, BRS: breast surgeon, NBS: surgeons other than breast surgery, JSMO: Japanese Society of Medical Oncology, BCMO: board-certified medical oncologist, BCGCO: board-certified general clinical oncologist
